# Therapeutic Potential of Controlled Delivery Systems in Asthma: Preclinical Development of Flavonoid-Based Treatments

**DOI:** 10.3390/pharmaceutics15010001

**Published:** 2022-12-20

**Authors:** Sergio M. Borghi, Tiago H. Zaninelli, Jéssica B. Carra, Olivia K. Heintz, Marcela M. Baracat, Sandra R. Georgetti, Fabiana T. M. C. Vicentini, Waldiceu A. Verri, Rubia Casagrande

**Affiliations:** 1 Department of Pathology, Center of Biological Sciences, Londrina State University, Londrina 86057-970, PR, Brazil; 2 Center for Research in Health Sciences, University of Northern Paraná, Londrina 86041-120, PR, Brazil; 3 Department of Chemistry, State University of Londrina, Londrina 86057-970, PR, Brazil; 4 Vascular Biology Program, Boston Children’s Hospital, Department of Surgery, Harvard Medical School, Boston, MA 02115, USA; 5 Department of Pharmaceutical Sciences, Center of Health Science, Londrina State University, Londrina 86038-440, PR, Brazil; 6 Department of Pharmaceutical Sciences, School of Pharmaceutical Sciences of Ribeirão Preto, Ribeirão Preto 14040-900, SP, Brazil

**Keywords:** controlled delivery systems, flavonoid, nanoparticles, asthma

## Abstract

Asthma is a chronic disease with increasing prevalence and incidence, manifested by allergic inflammatory reactions, and is life-threatening for patients with severe disease. Repetitive challenges with the allergens and limitation of treatment efficacy greatly dampens successful management of asthma. The adverse events related to several drugs currently used, such as corticosteroids and β-agonists, and the low rigorous adherence to preconized protocols likely compromises a more assertive therapy. Flavonoids represent a class of natural compounds with extraordinary antioxidant and anti-inflammatory properties, with their potential benefits already demonstrated for several diseases, including asthma. Advanced technology has been used in the pharmaceutical field to improve the efficacy and safety of drugs. Notably, there is also an increasing interest for the application of these techniques using natural products as active molecules. Flavones, flavonols, flavanones, and chalcones are examples of flavonoid compounds that were tested in controlled delivery systems for asthma treatment, and which achieved better treatment results in comparison to their free forms. This review aims to provide a comprehensive understanding of the development of novel controlled delivery systems to enhance the therapeutic potential of flavonoids as active molecules for asthma treatment.

## 1. Introduction

According to the World Health Organization (WHO), an estimated 262 million people were suffering from asthma in 2019, of which 455,000 cases resulted in patient death. Nowadays, this prevalence is around 300 million worldwide [1,2]. Economically, asthma-related costs and its social impact are overwhelming, varying according to the income of the countries [3]. When the direct and indirect effects are considered in association with uncontrolled asthma the cost burden reaches the exorbitant average value of USD 963.5 million solely in the US [4].

Asthma is a complex, heterogeneous, long-term condition that disrupts the physiology in the lung airways, characterized by distinct and recurrent pathogenic mechanisms including airway obstruction, hyperresponsiveness and inflammation. It is considered a major noncommunicable disease that is potentially life-threatening. Similar to many diseases, asthma also involves inherited genetic factors, environmental and lifestyle aspects [2,5]. Wheezing, dyspnea, and coughing are among the main symptoms of asthma; however, tightness in the chest, breathing difficulty and exercise symptoms may also occur. Nighttime awakening may be linked to a more severe clinical picture in adults [6,7]. This respiratory inflammation affects individuals of all ages and is the most frequent chronic illness found in children [2,5]. Importantly, the presentation of symptoms can vary by gender. Female asthma patients report more asthma symptoms and poorer disease control than male patients. As a result, there is a higher impact on daily activities and an increased number of visits to health professionals by female than male asthma patients [8].

Asthma is usually treated according to the symptom severity. Corticosteroids associated with bronchodilators such as short- or long-acting β-agonists (SABAs and LABAs, respectively), as well as leukotriene (LT) receptor antagonists (LTRAs), represent the reference choices for treatment options. For more severe cases, IgE- or Th2 cytokines-specific monoclonal antibodies are indicated [6,7]. Despite their efficacy, these medications present several undesirable side effects [6,9]. Therefore, given its socioeconomic and quality-of-life importance, alternative treatments with equivalent efficacy, novel targeted approaches and fewer adverse effects are desirable.

New possibilities of treatments for asthma include a group of molecules called flavonoids. They represent an important group of natural compounds with distinct phenolic structure [10,11]. They are broadly distributed in plants as secondary metabolites, being found in fruits and vegetables that compose human diet and have already demonstrated diversified biological properties that render flavonoids as potential treatments for various diseases. Literature supports the beneficial biological activities of flavonoids, which range from antioxidant, anti-inflammatory and analgesic to anti-infective, anticancer, hepatoprotective and neuroprotective effects [11,12]. A main difference with flavonoids in comparison to most current treatments in clinical use is that these polyphenols are multi-target drugs, opposed to the current single-target therapy approaches. By targeting multiple pathways, flavonoids do not abrogate one single pathway, but rather inhibit multiple processes avoiding side effects caused by excluding the physiological role of such targeted pathways. Other fields interested in flavonoids include the areas of functional foods and nutraceuticals sciences that have been growing [11,13,14,15].

As with many other classes of molecules, bioavailability is an issue for most flavonoids. In fact, this aspect represents a disadvantage for oral medicines, including the consumption of flavonoids from foods given the physiology of gastrointestinal tract and unsatisfactory absorbance and poor bioavailability of flavonoids [11,16,17]. Development of pharmaceutical forms to increment the activity and control the release of flavonoids is a possible strategy to circumvent this bioavailability issue. During the digestion process, the interaction of phenolic compounds with other molecules can be impaired. In this sense, the application of technology using optimization processes to preserve and/or boost the biological effects of flavonoid compounds might be essential to make full usage of their therapeutic potential [11,18]. Evidence supports that target delivery can make an inactive flavonoid into an active treatment [19]. Therefore, the development of novel therapies using nanotechnology represents an evolving field to achieve effective treatment for asthma patients. Controlled release can be achieved, for instance, by encapsulation technology in which active substances or their products are effectively coated by a structural complex or embedded in a matrix that protects it from negative external influences, allowing the controlled and targeted release of active substances in an environment with unfavorable conditions [18,20]. This process generates small capsules ranging from nanometers to several millimeters [21]. Examples of nanoparticle systems used for controlled delivery systems includes liposomes as well as polymeric and inorganic particles. Importantly, the efficacy of encapsulation may be affected by some factors during applied techniques. The finetuning of parameters for optimization includes, for example, the concentration of the polymer, the solubility of polymers in organic solvent, the solubility of organic solvent in water, ratio of the dispersion phase to the continuity phase, and solvent removal rate will determine the efficiency of the system for phenolic encapsulation [22,23]. These issues will be addressed in this review within the context of preclinical studies focusing on asthma models. 

**Figure 1:**
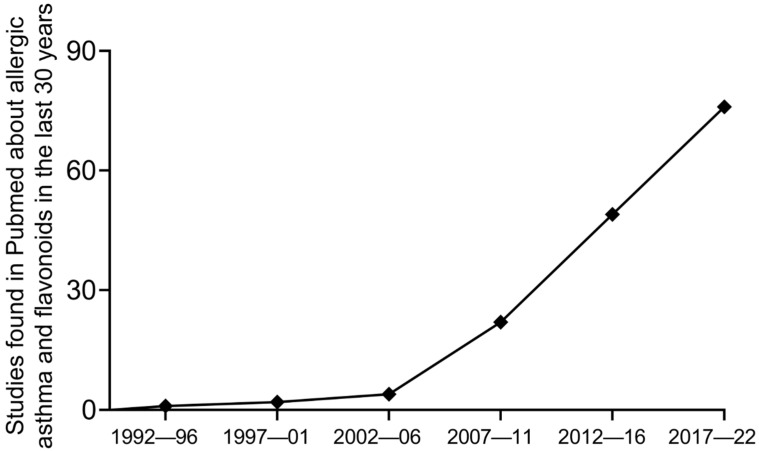
Original studies published on Pubmed in the last 30 years on preclinical asthma testing flavonoids. The keywords used for the search were “allergic asthma model and flavonoids”.

Figure 1 shows the growing number of publications on flavonoid and asthma in the last 30 years, which in addition to isolated compounds also involves plant extracts in which flavonoids constitute major active molecules. To our knowledge, there is no previous review article addressing the development of controlled delivery release systems to improve the activity of flavonoids in asthma; thus, this article focused on this literature gap. We reviewed the recent advances evaluating the effectiveness of controlled delivery systems applying flavonoids in the treatment of preclinical allergic asthma.

## 2. Asthma Physiopathology, Current Treatments, and Related Challenges

The understanding of the immunopathological mechanisms and complex heterogeneity underpinning asthma achieved great advances in recent decades. According to the Global Initiative for Asthma (GINA) Strategy Report from 2021, asthma is defined as a long-term and heterogeneous disease characterized by the history of respiratory symptoms, likely shortness of breath, wheezing, coughing, and chest tightness, which shows intensity variations over time [24]. Patients usually present infrequent expiratory airway flow limitation in association with hyperresponsiveness of the airways, which results from the response to the most varied stimuli such as exercise and inhaled irritants. Asthma is characterized by chronic inflammation and structural remodeling of the airways, with varied clinical presentations, treatment responsiveness, and natural manifestations during the patient’s lifetime [7]. 

The molecular phenotype of clinical aspects is strongly influenced by host–environmental factors. Importantly, the role of modern industrialization in asthma pathology is emerging, with the hypothesis of epithelial barrier breakdown continuing to gain strength [25]. The inflammatory profile observed in asthma patients may be driven by paucigranulocytic (low inflammatory cell counts in the sputum), eosinophilic, neutrophilic or a mixture of these cells, with the possibility of the profile changing over time [7,26]. Pathological features might be triggered by allergens (however, non-allergic asthma mechanisms remain poorly understood, and is often developed later in life), exercise, and occupational characteristics [7,26]. Highly allergic type 2 airway inflammation and non-allergic eosinophilic asthma are signalized by an immune cell infiltrate composed predominantly by eosinophils and Th2 lymphocytes, with moderate mucus production and preponderant airway smooth muscle mass. Th2 phenotype affects most adults and children, which in fact may be 50% or even more. On the other hand, non-eosinophilic asthma is characterized, as the name states, by few or lack of eosinophilic inflammatory infiltrate, as well as lessened mucus production and airway smooth muscle mass compared to highly allergic type 2 airway inflammation. Again, both adults and children are affected. The mixed granulocytic phenotype presents immune cell infiltrate composed of both eosinophils and neutrophils with increased mucus production and epithelial damage. Neutrophilic inflammatory asthma (Th1 and Th17) does not present eosinophils in the immune infiltrate, but rather features increased mucus production and epithelial damage associated with airway remodeling. Concomitant bacterial infections and corticoid down-modulation of the type 2 inflammation, which Th1, Th17 and ILC3 responses seem to underly, at least in part, the difference in immune response and variation in clinical presentation (for a detailed review, please see Papi et al., 2018) [26].

Allergen specific CD4^+^ T cells recruited to the airways in asthmatic patients are more sensitive to allergens than their equivalents in non-asthmatic controls. Asthmatic patients present higher type 2 cytokine release and consequently, greater type 2 inflammation than non-asthmatics exposed to the same antigen [27]. Airway epithelial cells under injury induced by allergens and/or microbes produce the alarmins Interleukin (IL)-25, IL-33 and thymic stromal lymphopoietin (TSLP), whose receptors are expressed by type 2 innate lymphoid cells (ILC2s) to also release type 2 cytokines (IL-5 and IL-13) and prostaglandin D_2_ (PGD_2_) [26,28]. This type 2 cytokine milieu is sufficient to stimulate antigen presentation by dendritic cells (DCs) to trigger an adaptive immune response such as the development of Th2 lymphocytes, which in turn produce large quantities of effector cytokines interleukin (IL)-4, IL-5 and IL-13 and others. Thus, ultimately leading to airway allergic sensitization [26,28] and characteristic type 2 inflammatory response with IgE release as well as eosinophil and mast cell recruitment and activation [29]. Mechanistically, IL-4 stimulates B cells to switch their immunoglobulin class production to IgE [30] while IL-5 release drives the survival and maturation of eosinophils [31,32]. Th2 cytokines are also found to induce epithelial changes and increased expression of eosinophils chemotactic factor eotaxin [28,33]. IL-13 contributes to increased periostatin expression in epithelial cells and modulates airway remodeling via goblet cells metaplasia, raising mucus hypersecretion and consequently increasing luminal narrowing and reducing forced expiratory volume [34,35]. Under IgE stimulation through FcεR1 high affinity receptors, mast cells acquire a profile in which tryptase and chymase levels increase as well as PGD_2_ and leukotrienes release. In severe asthma, there is an increase in mast cell numbers in the epithelium [28].

Asthma treatment can involve both non-pharmacological and pharmacological approaches. The dose of pharmacological treatment is adjusted for each asthmatic patient and should take into account not only the intended activity and efficacy, but also attempt to restrict the dose to the minimum to achieve disease management and at the same time reduce side effects [26]. Anti-inflammatory treatment represents the central axis for long-term asthma control. Quick-relief medications (described as SABAs) are recommended, generally, to obtain bronchodilation for patients with mild asthma, with reduced nocturnal symptoms and without risk for exacerbations. SABAs include inhaled β2-agonists albuterol, pirbuterol, and levalbuterol, and the anticholinergic ipratropium bromide (muscarinic antagonists) [6,26,36]. Adverse effects include headache, tachycardia, nervousness, and tremors. Intravenous magnesium sulphate together or not with SABAs or corticosteroids promotes bronchodilation in acute asthma. Systemic corticosteroids such as methylprednisolone represent an option for acute asthma and successive exacerbations [5]. Preventers or controller medications aim to treat underlying airway inflammation. Inhaled corticosteroids including fluticasone propionate, budesonide, mometasone, beclomethasone, and ciclesonide are often used as long-term control drugs in asthma treatment. They effectively provide daytime and nocturnal reduction in symptoms with optimized forced expiratory volume as well as decreased exacerbation episodes and death. Glucocorticoids target the activation of the pro-inflammatory transcription factor nuclear factor κB (NFκB). The potent inhibition of NFκB by glucocorticoids might occur via induction of IκB alpha inhibitory protein, which complexes with NFκB entrapping it inactive in cell cytoplasm. Moreover, glucocorticoid receptor (GR)-ligand interactions with glucocorticoids occur in the cytoplasm, and after conformational rearrangements, translocate to the nucleus, where GR-glucocorticoid complex binds to specific DNA sequences named glucocorticoid response elements (GRE) allowing induction of anti-inflammatory molecules. These cellular-mediated immunomodulatory effects of glucocorticoids and/or GR regulate the repression of pro-inflammatory mediators, including cytokine genes with pivotal roles in asthma, such as IL-4, IL-5, and IL-13 [37,38,39,40]. Adverse effects of inhaled corticosteroids described in the literature include upper airway infections, sinusitis, headache, and others [6]. Prolonged use of corticosteroids also results in immunosuppression, especially by sequestration of CD4^+^ T lymphocytes in the reticuloendothelial system and by targeting NFkB-dependent cytokine production [41]. This immunosuppression has clinical outcomes such as increase in susceptibility to mild and life-threatening infections, with a proportional linear increase in the risk with dose and duration of glucocorticoid therapy [42,43]. However, regular low doses of glucocorticoids in association with β2 agonists treatment attenuates exercise-triggered asthma, reduces the death outcomes and hospitalizations [26,44,45,46,47]. In cases in which exacerbations remain uncontrollable, long-acting β2-agonists or long-acting muscarinic antagonists, such as salmeterol, formoterol, vilanterol, and isoproterenol, and tiotropium bromide, respectively, can substitute SABAs for combination with inhaled corticosteroids [6,26]. They significantly improve forced expiratory volume [48,49,50]. Although considered full β2-agonists (different from partial agonists such as albuterol) and having superiority for therapeutic purposes when compared to partial agonists, formoterol, and isoproterenol, for example, are associated with an increased risk of adverse systemic effects [36].

Add-on therapies are recommended for severe asthma in which the control of symptoms is non-satisfactory, and when there is an association between disease aggravation and airway function limitation. There are receptor antagonists and synthesis inhibitors that target leukotrienes and cysteinyl leukotrienes, which are lipid mediators that play a crucial role in asthma pathology. These molecules function better as pretreatment to reduce asthma exacerbation episodes. Examples include the cysteinyl LTRAs zafirlukast and montelukast, and the inhibitor of leukotriene synthesis zileuton (a 5-lipoxygenase inhibitor) [51,52,53,54]. These drugs effectively protect bronchi against allergen challenges and attenuate bronchial remodeling, efficiently target daytime/nocturnal crisis, and improve forced expiratory volume [51,52,55,56]. However, their list of adverse effects includes headache, abdominal pain, dyspepsia, myalgia, mood changes and hepatotoxicity [6]. Theophylline (dimethylxanthine), a phosphodiesterase inhibitor, may also be added as a long-term medicine in uncontrolled asthma for adults [26,57]. Theophylline possesses both bronchodilator and anti-inflammatory effects. It is indicated for patients that show low responsiveness to inhaled corticosteroids with or without long-acting β2-agonists. Administration can be per oral for chronic treatment or intravenously for acute exacerbations [57,58]. Adverse effects of theophylline include nausea, vomiting and headache as well as cardiac decompensation and seizures, which occur when high concentrations are achieved in the plasma [57]. It is proposed that systemic corticosteroids should be used after approaches with leukotriene modifiers or theophylline are not effective, especially because steroids present a pallet of adverse effects [26]. In cases where the first therapeutic approach fails, the corticosteroids prednisone and methylprednisolone are commonly recommended options for oral treatment in asthma [6].

Monoclonal antibodies are an often-useful option for the treatment of severe asthma, as they can efficiently target several signaling pathways in type 2 inflammatory asthmatic reactions. Immunobiological compounds are engineered to act as antibody-based effectors or fusion proteins, to promote immune modulatory functions. Omalizumab blocks systemic IgE and is preconized for adults and children, in which treatment effects are specially observed as decreased exacerbation periods [59,60]. It presents adverse reactions such as the appearance of skin reactions at the infusion site, greater vulnerability to viral infections, headache, and anaphylaxis [6]. Other biologics used in severe asthma therapy include anti-cytokine medications, mepolizumab, reslizumab, and benralizumab, which are humanized anti-IL-5 monoclonal antibodies, targeting blood eosinophils. Anti-IL-5 effects include decreased severe asthma exacerbations together with minor positive effects in forced expiratory volume [61,62,63,64]. Importantly, mepolizumab dampens the need of steroids in asthmatic patients [65], and reslizumab showed superior efficacy in comparison to benralizumab in asthmatic patients with eosinophilic asthma [66]. Recently, the FDA approved tezepelumab, a monoclonal antibody against TSLP, for severe asthma, which reduces exacerbations in moderate-to-severe asthma and reduces systemic Th2 cytokines in uncontrolled severe asthma [67,68,69]. TSLP is an epithelial-derived, upstream molecule that activates multiple downstream inflammatory pathways and maintains several allergic responses and Th2 inflammation, acting, for example, in DCs and mast cells [70]. Therefore, these results highlight a promising treatment for severe asthma [28]. Strategies aiming to counteract IL-4 and IL-13 signaling in asthma are also ongoing, due to their fundamental roles in Th2 inflammatory reactions in asthma [71]. Dupilumab, a monoclonal antibody that targets both IL-4 and IL-13 receptors, demonstrated a safe profile in patients with uncontrolled persistent asthma in addition to lung function improvement. Most importantly, dupilumab provides disease relief even in cases of severe asthma already in a chronic phase that are unresponsive to medium/high doses of inhaled corticosteroids and long-acting β2-agonists [72]. In 2018, dupilimab received FDA approval as an add-on therapy for asthmatic patients with moderate-to-severe disease, characterized by eosinophilic phenotype or with oral corticosteroid dependence. Adverse reactions include injection site hypersensitivity, oropharyngeal pain, and eye and eyelid inflammation. Lebrikizumab is a monoclonal antibody that avoids IL-13 actions since it binds to the cytokine with high affinity, thus inhibiting the cytokine receptor activation. The FDA granted lebrikizumab fast-track designation for atopic dermatitis in 2019. In asthma trials it reduces airway remodeling (subepithelial fibrosis) and satisfactorily attenuates the rate of exacerbations, and ameliorates lung function in uncontrolled asthmatic patients, especially those with higher levels of periostatin [73,74,75]. In this sense, lebrikizumab is an emerging biological compound with great application potential for uncontrolled asthma. 

**Figure 2:**
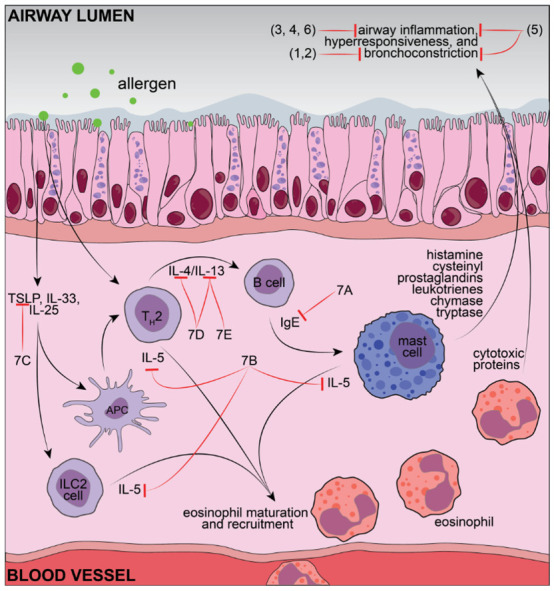
Th2 inflammation in response to allergens in asthma and targets of drugs currently used in the treatment of allergic asthma for humans: (1) Short-acting bronchodilators (β2-agonists; albuterol, pirbuterol, and levalbuterol) and antimuscarinics (ipratropium bromide) inhibit bronchoconstriction; (2) Long-acting bronchodilators (β2-agonists; salmeterol, formoterol, vilanterol, and isoproterenol) and muscarinic antagonists (tiotropium bromide) inhibit bronchoconstriction; (3) Inhaled corticosteroids (fluticasone propionate, budesonide, mometasone, beclomethasone, and ciclesonide) inhibit inflammatory response; (4) Leukotriene modifiers (zafirlukast, montelukast, and zileuton) inhibit hyperresponsiveness and inflammatory response; (5) Theophylline inhibits bronchoconstriction and inflammatory response; (6) Systemic corticosteroids (prednisone and methylprednisolone) inhibit inflammatory response; (7) Monoclonal antibodies (7A) omalizumab (anti-IgE), (7B) mepolizumab, reslizumab, and benralizumab (anti-IL-5), (7C) tezepelumab (anti-TSLP), (7D) dupilumab (anti -IL-4/IL-13), and (7E) lebrikizumab (anti-IL-13; not approved for asthma so far) inhibit specific signaling pathways related to Th2 inflammatory response. Figure was designed in Adobe Illustrator 2023 (Adobe©, San Jose, CA, USA).

Finally, it is important to mention that some barriers and paradoxes directly affect asthma management. The poor adherence to the therapies represents the fundamental problem for asthma treatment strategies. This is clearly observed when controller therapy is proposed to the patients [76,77]. In general, the use of inhaled drugs (e.g., corticosteroids) is a choice only when asthma symptoms are perceived. Accordingly, when no symptoms of exacerbations are perceived, patients interpret that there is no need for their use [26,76]. Nonetheless, when asthma symptoms aggravate, patients prefer to use reliver therapies (e.g., SABAs), a common scenario favoring SABAs overuse, which in turn might increase the risk of death by the exorbitant use of β-agonists [45,47]. There is also concern about oral corticosteroids in asthma treatment. This kind of fear occurs because both oral- and short-term use corticosteroids are related to the adverse events mentioned above, which are proportional to the exposure to corticosteroid [78,79,80]. For these reasons, alternative treatment options that provide less adverse effects, equivalent or superior efficacy to current approaches, and consequently, better adherence rates are needed. Additionally, public policies on raising awareness about the importance of strictly following the treatment recommendation (time of use and dose of drugs, etc.) and encouraging the treatment of comorbidities and behavior changes are also necessary. Figure 2 shows the schematic representation of asthma pathophysiology, and simultaneously, the respective targets of current treatments available for asthma as discussed in this section.

## 3. Flavonoids: Chemistry and Biological Actions

**Figure 3:**
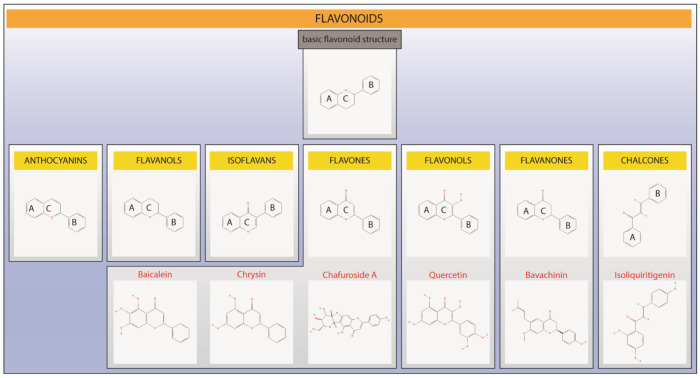
Basic structure of flavonoids, main classes, and examples (in red) of molecules from classes tested using nanotechnology in asthma models. Figure was designed in Adobe Illustrator 2023 (Adobe©, San Jose, CA, USA). Chemical structures were downloaded from PubChem (pubchem.ncbi.nlm.nih.gov).

The interest in natural compounds obtained from various medicinal plants and food sources has been increasing significantly in the past decades, especially by the pharmaceutical industry that aims to commercialize these products with improved bioavailability. Representing one of the most prominent and diversified groups among natural products, flavonoids constitute a major group of natural substances composed by chemical phenolic ring structures, which are, in fact, responsible for most biological properties of this group [13]. Flavonoids are found in the plant kingdom, in bark, roots, stems, flowers, fruits, vegetables, and grains. Thus, flavonoids are a regular part of human diet [10,13]. For a more detailed review about flavonoid food sources, please see Kozłowska and Szostak-Węgierek, 2017 [81]. Flavonoids can be divided into classes and share a basic chemical structure. There is a core flavonoid nucleus composed of three phenolic rings designated A, B, and C. Fifteen-carbon skeleton and two benzene rings connected through a heterocyclic pyrane ring, carrying one or more hydroxyl groups may be found in nature, in aglycone or glycoside forms (Figure 3) [82,83]. Importantly, the location of the hydroxyl group determines the biochemical mechanistic action of diverse flavonoids, which in many cases might act as multi-target compounds [13]. Chemical structures of flavonoids and their metabolites may also undergo several reactions, including hydrogenation, methylation, malonylation, sulfonation and glycosylation, thus forming a great variety of flavonoids with different hydrophilicity/hydrophobicity, absorption, and bioactivity [84,85]. They are conjugated to acid or sugar moieties and present as oligomers, therefore, generating polymers referred to as tannins [86]. We suggest prior publications to deeper analyze flavonoid chemistry [11,13].

The bioactivity and the expected health effects of flavonoids will depend on the route of administration, phytochemical properties (molecular size, lipophilicity, water solubility, glycoside or aglycone structure, etc.), adequate absorption, and bioavailability of the different compounds. Remarkably, the low bioavailability after oral administration of flavonoids has been a concern as it might substantially hinder their beneficial properties. For this reason, attempts to optimize their bioavailability are continuously in progress [13,83,87,88]. Flavonoids can be absorbed in the stomach and small intestine, via active transport or passive diffusion, or in a minor quantity absorbed and metabolized by intestinal microbiota [89]. Flavonoid excretion occurs via feces or urine as a result of conjugation by glucuronidation or sulfation in the liver cells, or even metabolization into minor phenolics; no free aglycones are observed in plasma or urine for most flavonoids [83,90].

The main classes of flavonoids include flavones (e.g., luteolin, apigenin, wogonin, baicalein, vitexin, and chrysin), flavanols (e.g., flavan-3-ols; e.g., catechin, gallocatechin, epigallocatechin, epicatechin, and epicatechingallate), flavanones (e.g., hesperidin, naringin, naringenin, taxifolin, and eriodyctiol), flavonols (e.g., myricetin, kaempferol, silymarin, quercetin, rutin, and fisetin), anthocyanins (e.g., apigenidin, pelargonidin, cyanidin, delphinidin, and peonidin), isoflavones (e.g., formononetin, biochanin A, genistein, daidzein, and glycetein), and chalcones (e.g., naringenin chalcone, trans-chalcone, butein, 2′-4′-dihydrochalcone, and hesperidin methyl chalcone) [10,13,86,89]. The classes may vary according to the degree of oxidation and profile of the C ring replacement [13,83]. Figure 3 presents the basic chemical structure of flavonoids as well as the chemical structures of the main classes together with examples. A huge number of biological properties of flavonoids are described in the literature, which range from anti-inflammatory, immunomodulation, analgesic, anti-cancer, anti-angiogenic, and anti-cholinesterase to antimicrobial actions. Nevertheless, their antioxidant activity is the most described effect of almost all flavonoid classes and the structure–activity relationship to achieve this activity is the best described effect [13,83]. The antioxidant mechanistic action of flavonoids involves the attenuation of free radical generation (e.g., by enzymes inhibition or chelating actions), free radical elimination, and optimization of antioxidant defenses (e.g., by raising glutathione reduced levels or stimulating antioxidant enzymes) [91,92]. For a deeper understanding of structure/activity relationship of flavonoids regarding their antioxidant activity, we suggest some previous publications [11,13].

The anti-inflammatory activity of flavonoids is also a result of their antioxidant activity, since reactive oxygen and nitrogen species participate in inflammation not only as molecules that potentially cause cellular and tissue damage, but also as signaling molecules governing the inflammatory response [11,13,93,94]. However, the anti-inflammatory effect of flavonoids may also occur via pro-inflammatory cytokine reduction, as they have been demonstrated to target transcription factors such as NFκB that orchestrates the production of pro-inflammatory cytokines which act as downstream effector peptides of this pathway in asthma pathology [13,89,95,96]. Additional important activities include the inhibition of cyclooxygenase 2, an inducible enzyme that metabolizes arachidonic acid producing PGH_2_, an intermediary that will undergo further metabolization generating prostaglandins and thromboxane depending on the isomerases present in each cell type [97]. Flavonoids can also inhibit the expression of inducible nitric oxide synthase (iNOS) that produces great amounts of nitric oxide during inflammation with functions of regulating vasodilatation, microbial killing, and cellular damage [10,89,95,98]. The induction of the nuclear factor erythroid 2–related factor 2 (Nrf2) is also a notable mechanism of flavonoids anti-inflammatory effects. Nrf2 up-regulates the expression of heme oxygenase-1 (HO-1), NAD(P)H:quinone oxidoreductase-1 (NQO1), glutathione peroxidase (GPx), glutamate cysteine ligase (GCL), superoxide dismutase (SOD), catalase, glutathione S-transferase (GST), and thioredoxin UDP-glucuronosyltransferase; thus, it leads to antioxidant and detoxifying effects [99]. Nrf2 also reduces the activation of NFkB diminishing inflammation [13,89,99,100]. Acting as anti-inflammatory agents, flavonoids also inhibit intercellular adhesion molecule-1 (ICAM-1), vascular cell adhesion molecule-1 (VCAM-1), endothelial cell adhesion molecule-1 platelet (PECAM-1), and E-selectin on endothelial cells, as well as the transcription factors mitogen-activated protein kinase (MAPK) and signal transducer and activator of transcription 3 (STAT3) pathways [13,89].

Importantly, flavonoids were shown to reduce type 2 inflammation physiopathological mechanisms [89,101]. Specifically on allergic inflammatory reactions in the context of asthma, flavonoids can reduce NFκB activation (e.g., naringenin, sakuranetin, kaempferol, fisetin, and rutin) [102,103,104,105,106], the reactivity of mast cells and basophils in response to allergens (e.g., flavanone, luteolin, baicalein, kaempferol, apigenin, fisetin, myricetin, quercetin, and rutin) [106,107,108,109,110], leukotriene synthesis (e.g., kaempferol, quercetin, and rutin) [111,112,113], Th2 cytokines (e.g., fisetin, luteolin, apigenin, quercetin, hesperidin, and naringenin) [110,114,115,116,117,118,119,120,121], the differentiation of naïve CD4^+^ T cells into effector cells via inhibition of the aryl hydrocarbon receptor signaling (AhR; AhR agonists induce pathologic aberrant T cells responses, particularly in Th2/Th17 pattern, and consequently, inflammation) after stimulation by toxic and biological agents of many aromatic environmental pollutants (e.g., apigenin, luteolin, and quercetin) [122], CD40 ligand expression (required for B cells production of IgE, and other high-affinity IgE receptor-expressing cells) (e.g., luteolin) [123], as well as IgE-dependent inflammation and/or IgE-related allergic reactions (e.g., luteolin, quercetin, apigenin, baicalein, narirutin, and naringenin) [105,117,121,124,125]. Chemically, the presence of a hydroxyl group at the 3′ and/or 4′ positions of the B-ring is fundamental for anti-inflammatory activities for some flavonoids [126,127]. Therefore, considering the crucial roles of the NFκB pathway, eicosanoids, oxidative stress, and Th2 signaling in asthma, and the resultant effective modulatory actions in the inhibition of these, flavonoids represent a powerful therapeutic tool for the management of asthma.

To this end, it is important to note that as flavonoids are generally described to possess low bioavailability (discussed earlier in this section), their efficacy in asthma treatment might be improved by developing pharmaceutical formulations, in which controlled-release systems may bring countless advantages. To address this issue, studies evaluating the effectiveness and mechanistic actions of flavonoids using this technology for asthma control are discussed in the next section. 

## 4. Controlled Flavonoid Release Systems in Asthma Treatment

Controlled drug delivery systems are designed to have characteristics of drug release time and/or location planned to achieve certain pharmacological and therapeutic goals. They are widely used because they maximize the protection of the active compounds and therapeutic effects, lower dose fluctuation in the body and raise drug bioavailability, reduce side effects and the frequency of drug administration [128,129,130]. These systems include many types of formulations, such as nanoparticles (nanocapsules, nanospheres, and nanocrystals), microparticles, liposomes, nano and microemulsions, liquid crystals, polymeric films, and gels (nanogels, organogels, and hydrogels), among others [131,132,133,134,135,136]. 

In recent years, a few research groups have evaluated the use of controlled drug delivery systems to release flavonoids in asthma treatment. They commonly use methods applied to improve the bioavailability of hydrophobic drugs like emulsification (diffusion, self-assembly, and solvent evaporation), coacervation, solvent displacement (nanoprecipitation method), and ultrasonication to obtain them. In the emulsification-diffusion method, the lipid matrix is dissolved in water with a surfactant, then more water is added to the system which causes drug diffusion into the oil phase, forming the emulsion [137]. Self-emulsification is a method in which oil, surfactant, and hydrophilic co-surfactant self-organize to form micro or nano emulsions [138]. In the emulsification-solvent evaporation method, the polymer and the active compound are dissolved in a volatile organic solvent, after which water and a stabilizer are added to the oil phase to form an o/w emulsion; afterward, the solvent is evaporated [139] and the micro/nanoparticles are obtained. The coacervation method is based on the precipitation of colloids (polymer with drug) formed by a change in pH, temperature, or the addition of a non-solvent as salt; it could be simple, using one type of polymer, or complex, using two or more polymers with charged polyelectrolytes [140]. The nanoprecipitation method (solvent displacement) consists of dissolving the polymer with the drug in an intermediate polarity solvent and then adding water while stirring. The organic solvent displaces to the water and the water-insoluble polymer precipitates [141]. Lastly, ultrasonication can be used to produce nanocrystals by reducing and homogenizing the particles by high-frequency sound waves [142]. In fact, the use of nanotechnology for chronic respiratory diseases such as asthma was recently proposed, with the intension of providing slower metabolism and sustained release of the compounds [143]. Next, preclinical studies applying nanostructured systems for asthma management are discussed.

The flavonol quercetin (C_15_H_10_O_7_; Figure 3) is one of the major dietary flavonoids and is approved by the FDA for human use [144]. The antioxidant property of quercetin is considered equivalent to that presented by ascorbic acid. The current understanding is that its potent antioxidant actions are due to the high content of hydroxyl groups associated with the fact that hydroxyl groups in C-ring may form a resonance stabilized quinone structure [145]. However, quercetin modifications such as glycosylation or methylation may hamper the antioxidant capacity of this flavonol [146]. Quercetin may present toxicity at high doses due to the generation of pro-oxidant effects; however, this does not seem to occur under therapeutic doses [147]. Quercetin was tested in two studies applying nanostructured systems. In the first study, Rogerio et al. (2010) investigated the anti-inflammatory activity of quercetin in two pharmaceutical forms, a microemulsion (o/w), and a suspension, systems. The microemulsion was prepared by the hot solvent diffusion method, using lecithin, castor oil, and Solutol HS15^®^ (0.02:0.2:1). For the suspension, quercetin was dispersed in aqueous carboxymethylcellulose dispersion (0.5%). They evaluated these formulations in an ovalbumin (OVA)-induced model, using female BALB/c mice and male Wistar rats. For that, the microemulsion (3 or 10 mg/kg) or the suspension (10 mg/kg) were given orally by gavage from the 18th to the 22nd day after the first inoculation with OVA. The results of suspension did not show anti-inflammatory activity or the presence of quercetin in the animals’ blood, while the results of microemulsion (10 mg/kg) showed evidence of quercetin in the blood of animals and reduced the eosinophil recruitment, IL-4, and IL-5 levels, but not LTB_4_ or interferon-γ (IFN-γ) in the bronchoalveolar lavage fluid, together with reduced P-selectin expression and mucus secretion in the lung. An interesting finding was the inhibition of NFκB pro-inflammatory subunit p65 and of mucus production in a manner equivalent to dexamethasone in lung tissue [148]. Gupta et al. (2016) obtained nanocrystals applying ultrasonication in a mixture of quercetin and Tween 80. They evaluated the anti-asthmatic activity of this compound by exposing female BALB/c mice to OVA and injecting 1 mg/kg of quercetin nanocrystals intraperitoneally. They concluded that the transformation into nanocrystals improves the bioavailability and solubility of quercetin in water, in addition to reducing OVA-induced Th2 cytokines IL-4 and IL-5 levels, and IgE production, as well as attenuating the activation of mast cells, and improving the control for allergic asthma [149]. The flavone chafuroside A (C_21_H_18_O_9_; Figure 3) is a flavone C-glycosides first isolated from oolong tea leaves, which possesses an unusual structural characteristic in the ether connection of C2″-hydroxyl group of the mannose and the aglycone compounds [150]. Structure–activity studies demonstrated that the flavone moiety of chafuroside A is curved, which may positively interfere in its pharmacological activities [150]. Chafuroside A was reported to be safe in mice models of colon cancer from 20 to 25 parts per million (p.p.m.) dose range variation [151]. Onoue et al. (2012) prepared a self-assembled micellar formulation of chafuroside A, mixing this compound with PEG400, propylene glycol, Tween 80, and ethanol (1:1:1:1). They tested the oral formulation (0.1 mg CFA/animal) and the free compound (0.1, 0.5, or 1.0 mg/animal) at male Sprague–Dawley rats, using OVA-induced asthma model, over seven days. The self-assembled micellar system demonstrated potent anti-inflammatory activity, showing reduced infiltration of macrophages and eosinophils when compared to the free compound at all doses tested. Regarding the number of neutrophils, the results were similar to the dose of 10 mg for the free compound. Thus, this delivery drug system for flavonoids looks promising for asthma treatment [152].

Bavachinin (C_21_H_22_O_4_; Figure 3) is a prenylated flavonoid from the flavanone class [153]. It is recognized as an important peroxisome proliferator-activated-γ (PPAR-γ) agonist. Docking studies demonstrated that this agonistic activity is due to the presence of an isopentenyl group at the C6 position and a methoxyl group at the C7 position [154]. Evidence supports that bavachinin can induce hepatotoxicity via reactive oxygen species (ROS), which also triggers the activation of mitogen-activated protein kinases c-Jun N-terminal kinase (JNK) and p38. However, it is important to mention that this effect occurs in vitro in HepaRG cells with an IC_50_ of 14.28 μM in a 24 h exposition [155]. As a general assumption, drugs under in vitro screening are of potential drugability with high potency/excellent activity when the IC_50_ is up to 1 μM; when the IC_50_ is between 1 and 20 μM the compound is considered to have good activity alone [156]. Considering that bavachinin can only achieve PPAR-γ agonistic function, its main mechanism of action, at an EC_50_ value of 0.74 μM for PPAR-γ, it is likely that this toxic side effect will occur at higher doses of bavachinin or when there is a preexisting background of liver disease or co-consumption of other molecules that affect liver function and metabolism [157]. Wang et al. (2018) produced bavachinin-loaded nanoparticles by an emulsion-solvent evaporation technique. For that, they mixed the active poly(ethylene glycol)-poly(lactic acid-co-glycolic acid) (PEG-PLGA) (1:10) with chloroform, and then added into an aqueous solution of 2% polyvinyl alcohol. The dispersion was sonicated and stirred to remove the solvent. The nanoparticles (50 mg/kg) were tested orally in female BALB/c using an OVA model to evaluate anti-asthmatic activity of the system. The results showed that the bavachinin-loaded nanoparticles present significant results upon the reduction in Th2 cytokines mRNA expression (IL-4, IL-5, and IL-13) as well as IL-4 protein levels and lung histopathology [158]. In another study, bavachinin-loaded nanospheres were also evaluated as an anti-asthmatic compound. This flavonoid of the flavanone class presented increased anti-inflammatory activity in this oral delivery system, as well as presenting lower toxicity than steroids. The results showed a significant reduction in Th2 cytokines. Moreover, lung histopathology was satisfactorily improved in the treatment group, since it efficiently targeted the airway remodeling and the infiltration of immune cells [159]. 

Chrysin (C_15_H_10_O_4_; Figure 3), a flavone plant-derived flavonoid, is described as presenting several pharmacological effects. Similar to bavachinin, chrysin is proposed to possess agonistic effects upon PPAR-γ activity, which results in the downregulation of cyclooxygenase 2 (COX-2) activity, and consequently, reduced production of prostaglandin E_2_ [160]. Chrysin flavone analogs are composed by 6- or 8-substituent groups at the A ring of the flavone structures, which is not found in the chrysin structure; this may explain the enhanced inhibitory effects of these analogs when compared to chrysin [160]. For this reason, it is believed that chemical modifications of this flavone may enhance its pharmacological activity [161]. A recent study evaluated the toxicological effects of chrysin and identified its LD_50_ to be 4350 mg/kg in rats, whilst at the dose of 500 mg/kg no observable adverse effects were described regardless of gender. Thus, it is likely that toxic effects are observed only at high doses that are beyond the therapeutic range [162]. Roy et al. (2020) studied the chrysin -loaded PLGA using the solvent displacement method. For that, they prepared the dispersion of the compound and the polymer (1:10) and precipitated the nanoparticles with an aqueous solution of 1% polyvinyl alcohol overnight. In vitro investigations showed the slow and sustained release of the nanosized compound in the developed system. This formulation at the concentration of 50 mg of chrysin/kg was tested orally in male BALB/C mice with OVA-induced allergic asthma. The results showed that the nanoparticles of this compound can attenuate the inflammatory process of allergic asthma, as observed by dramatic decreases in macrophage, granulocyte and lymphocyte counts and Th2 cytokines (IL-4, IL-5, and IL-13) in bronchoalveolar lavage fluid, as well as in serum IgE and lung histopathology equivalent to dexamethasone, while the free active form (in the same dose) did not present the same positive results. Interestingly, chrysin nanoparticles suppressed toll-like receptor/NFκB/ NLR family pyrin domain containing 3 (NLRP3) inflammasome signaling in lung tissue, in an equivalent efficacy to dexamethasone and superior efficacy to the chrysin non-nanosized format, without inducing hepatotoxicity for up to 30 days after its use. These results highlight the potential applicability of chrysin nanoparticles for the treatment of asthma [163]. The chalcone-type flavonoid isoliquiritigenin (C_15_H_12_O_4_; Figure 3) is one of the components of Licorice (*Radix glycyrrhizae*). Several pharmacological properties are described for isoliquiritigenin [164]. Studies focusing on structure–activity relationship evidenced that isoliquiritigenin, and specially its derivatives, present inhibitory activities upon amyloid beta protein aggregation, where, in two of its most potent inhibitory derivatives, a 4-substituted side chain of N-methylpiperazin-1-yl and morpholine group in A ring were present. Moreover, regarding its anti-inflammatory actions, the inhibitory effect upon 5-lypoxygenase (5-LO) enzyme was attributed to a 4-substituted side chain of N-methylpiperazin-1-yl, and piperidine and morpholine group in A ring, which displayed the best inhibitory activity [164]. Finally, isoliquiritigenin (10–200 µM) presents toxic effects and induced necroptosis in the human neuroblastoma cell line SH-SY5Y [165], whereas its derivatives (0–100 µM) were described as presenting lower cytotoxicity in the same cell line [164]. However, as for the other flavonoids mentioned above, the toxic concentration range is higher than the drugable concentration range [156]. Further, isoliquiritigenin achieves maximal inhibition of lipopolysaccharide (LPS)-induced production of inflammatory molecules by Raw 264.7 macrophages at a concentration of 1.6 µM, meaning that the toxic concentration range is higher than the pharmacologically relevant concentration range [166]. Cao et al. (2020) produced isoliquiritigenin self-nanoemulsified using ethyl oleate, Tween 80, and PEG400 (3:6:1) sonicated for 10 min and kept under stirring overnight. The OVA-induced asthma model was applied in female BALB/c Sprague–Dawley rats to test these formulations with two doses of isoliquiritigenin (twice 5 mg/kg/day and twice 10 mg/kg/day), free isoliquiritigenin (twice 10 mg/kg/ day), and dexamethasone (twice 0.25 mg/kg/day) intragastrically for four weeks. The results showed that the self-nanoemulsifying isoliquiritigenin inhibited eotaxin-1 production in human lung fibroblast lineage (HFL-1). In vivo, improved oral bioavailability of the flavonoid was observed as well as reduced inflammatory cell infiltrate (eosinophils, neutrophils, and lymphocytes), IL-4, and IL-5; additionally, they observed an increase in IFN-γ levels in bronchoalveolar lavage fluid, diminished lung histopathology, and serum IgE as much as dexamethasone and better than free isoliquiritigenin [167].

The flavone baicalein (C_15_H_10_O_5_; Figure 3) is a natural antioxidant and antibacterial, with biological activities attributed to different substituents connected at B-ring and a higher number of hydroxyl groups [168]. Moreover, through its hydroxyl groups and B ring, baicalein blocks the interaction of human TSLP and its receptor in a dose-dependent manner [169]. In a randomized trial, single oral doses between 100 and 2800 mg of baicalein were safe and well tolerated by heathy subjects, with mild adverse events including abdominal distention, blood leukopenia and others, not exceeding 4% of the study participants [170]. Baicalein was also tested in the OVA-induced allergic asthma model. The authors compared two baicalein-nanoparticles, baicalein loaded nanoparticles (L-B-NP) and baicalein encapsulate nanoparticles (E-B-NP) using an inhalation method (1 mg/mL for 30 min/day). The first one was obtained by the simple coacervation method using chitosan as polymer and cinnamaldehyde as crosslinker. The cationic nanoparticles (the second one) were obtained by emulsification with solvent evaporation, using glyceryl monooleate as the lipid phase, poloxamer 407 as a stabilizer, gelucire 44/14 as an absorption enhancer, and trimethyl chitosan as cationic material. Both formulations were tested via inhalation (1 mg/mL for 30 min/day) by nebulizer in female BALB/c mice. The researchers concluded that both nanoparticles can be used as anti-asthmatics, but in different ways. Both treatments inhibited IL-5 levels in bronchoalveolar lavage fluid. The L-B-NP had a better controlled-release than E-B-NP, can reduce mucus secretion in the airway, and was better to control airway hyperresponsiveness; the E-B-NP was more effective at controlling airway inflammation. Therefore, L-B-NP is more efficient as a short-acting treatment while E-B-NP is better in as a long-acting treatment [171]. Table 1 summarizes the current preclinical evidence on controlled delivery systems testing flavonoids on asthma. 

Altogether, these preclinical data highlight that controlled drug delivery systems can be applied as novel flavonoid-based treatments aiming to advance the management of asthma patients. Moreover, clinical trials are needed to guarantee the efficacy and safety of these systems in humans.

## 5. Perspectives of Novel Formulations Containing Flavonoids for the Treatment of Asthma, and Improving Pre-Clinical Data towards Successful Human Translation

The use of flavonoids in the treatment of asthma using controlled-release systems is a path that still has much to evolve. Several formulations are used for flavonoids and could be used for the treatment of asthma. One important point in experimental design is that comparison with the free flavonoids is essential to determine whether there is some therapeutic improvement by the controlled drug delivery system. Improvement can be achieved by reducing the flavonoid dose, time to start activity and/or duration of activity. To this end, comparison between controlled drug delivery system and free drug is essential. Liquid crystals, polymeric films, nanogels, organogels, and hydrogels could be used in the transdermal route. This is not a common route of administration for the current asthma treatments; however, β-blockers can be administered via the transdermal route, for instance, to treat hypertension [172]. This indicates that transdermal route could be a novel approach to obtain continuous and prolonged release of flavonoids achieving systemic dosage [173]. Of note, transdermal administration avoids the liver metabolization before biological activity and gastrointestinal irritation [174]. Some flavonoids are both active molecules and pro-drugs [175], which means that the transdermal route of administration can, in theory, prolong the therapeutic effect of flavonoids by making a two-phase effect possible which would initially depend on the non-metabolized flavonoid and afterwards on the flavonoid active metabolites.

Liposomes, micelles, microemulsions, nanoemulsions, nanoparticles and nanospheres are active by per oral and intranasal routes [176,177,178]; however, only one study developed an emulsified nanoparticle for intranasal administration (Table 1). It is relatively common in asthma treatment to have approaches such as oral administration that reach higher lung or systemic levels [179]. As asthma therapy is chronic, systemic administration is avoided due to the side effects of treatments with, for instance, corticoids [26,76]. However, if the active dose of flavonoid does not induce side effects, per oral administration would be the most beneficial choice due to the easiness of administration and low cost to administer. Intranasal or inhalation are appealing routes of administration and used in the clinical practice of asthma treatment. They are intended to have a local effect and low systemic side effects [5,26,180]. Another point is to define whether flavonoids have effects against other asthma symptoms that are not solely inflammation parameters. For instance, do flavonoids reduce bronchoconstriction? If this is the case, fast-releasing formulations could be used for inhalation and intranasal administration. Evidence indicates some flavonoids (e.g., quercetin, chrysin, hispidulin, catechin, chrysoeriol, and isoliquiritigenin) reduce bronchoconstriction [181,182,183,184,185,186]. All these possible controlled drug release systems should present biocompatibility with the route of administration and can be designed to have a faster or slower release depending on the flavonoid biological mechanism of action in asthma. Consequently, this seems a field largely unexplored using flavonoids as active molecules.

**Figure 4:**
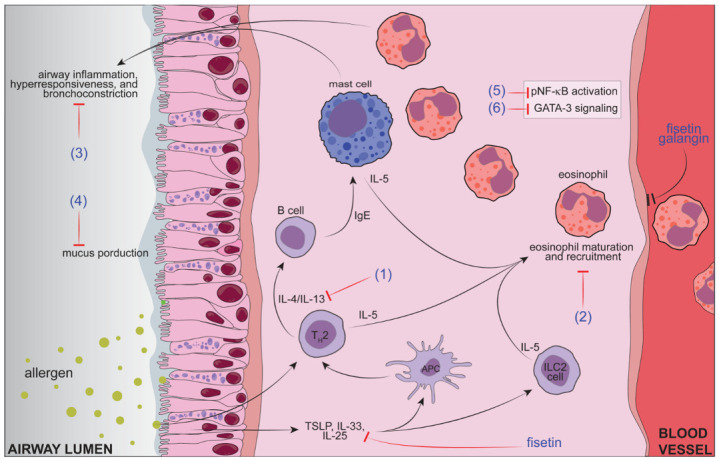
Mechanisms of flavonoids tested in their free forms in allergic asthma models: (1) Naringenin, kaempferol fisetin, licochalcone A, luteolin, apigenin, hesperidin, sakuranetin, naringenin chalcone, genistein, isoquercitrin, and sulfuretin inhibit Th2 cytokines; (2) Naringenin, kaempferol fisetin, licochalcone A, rutin, apigenin, hesperidin, sakuranetin, naringenin chalcone, genistein, isoquercitrin, galangin, and sulfuretin inhibit leukocyte recruitment, especially eosinophils; (3) Kaempferol, fisetin, licochalcone A, luteolin, rutin, apigenin, hesperidin, sakuranetin, naringenin chalcone, and sulfuretin inhibit airway hyperresponsiveness; (4) Kaempferol, fisetin, luteolin, and galangin inhibit mucus production; (5) Fisetin, sakuranetin, and sulfuretin inhibit NFkB (p65) activation; (6) Fisetin, apigenin, hesperidin, and genistein inhibit GATA-3 signaling. Fisetin inhibits TSLP. Fisetin and galangin inhibit adhesion molecules in endothelial cells. Figure was designed in Adobe Illustrator 2023 (Adobe©, San Jose, CA, EUA).

On the other hand, flavonoids have been widely studied for allergic asthma (OVA-induced, for example) in preclinical models (e.g., mice, rats, and guinea pigs). The literature displays papers on several flavonoids in their free forms (not in pharmaceutical formulations), such as naringenin [187], kaempferol [188,189], fisetin [118,190], licochalcone A [191], luteolin [192,193], rutin [112], apigenin [117,194], hesperidin [120], sakuranetin [102], naringenin chalcone [195], and others [196,197,198,199]. Thus, considering that data regarding a superior efficacy of controlled delivery systems than free forms (most papers compiled in Table 1), discussed above, it is possible to hypothesize that applying these technologies might improve therapeutic results for asthma management for those flavonoids whose activity in asthma has been demonstrated. Figure 4 highlights the described anti-asthmatic effects of flavonoids tested in their free form in models of allergic asthma.

Targeting NFkB activation and activity is the major mechanism of action of corticoids. The problem is that with this activity comes undesirable side effects related to the hormonal activities of corticoids [41,42,43]. In this sense, molecules that target NFkB activation and activity are promising strategies in asthma if they present lessened side effects compared to corticoids [89,143,200]. Lacking the hormonal side effects of corticoids would be a possible improvement [143,200]. Some flavonoids bind to corticoid receptors with antagonist functions (e.g., 5-hydroxyflavone) and others do not bind to corticoid receptors [101,201]. With both types of flavonoids (antagonize corticoid receptors or not) there will not be side effects similar to those caused by high corticoid levels achieved during systemic treatment compared to physiological concentration. Table 2 summarizes the current preclinical data on the activity of flavonoids in asthma models. Note that some of these flavonoids reduce asthma inflammation by reducing NFkB activation or activity. For some flavonoids this mechanism was not investigated (Table 2). 

The available literature on asthma models demonstrates that many animal species are used to study the mechanisms of asthma and possible treatments for the disease, including mouse, rats, guinea pigs, cats, and primates, among others, and different routes of therapeutic administrations. Mouse models represent the majority of studies investigating the airway inflammatory response to allergens [202]. Although it is important to recognize that great advances have been made with preclinical studies of asthma, notably disease models, they generally have some limitations since they are not identical to human disease. Human heterogeneity of disease manifestation (clinical presentations, physiological criteria and environmental stimulants, and biomarkers to identify distinct endotypes) suggests that applying varied models of disease would also be desirable [202,203,204,205,206]. Another issue is that asthma is artificially induced in mice. This leads to some disadvantages for mouse models, such as the non-observation of the non-physiological late-phase bronchoconstriction, a distinct profile of lung inflammation relative to humans, and absence of allergen-induced chronicity and tolerance induced by allergen exposure [202]. It would be desirable for preclinical studies aiming to mimic asthma pathology in humans to succeed in both sensitization and challenge phases [207]. Asthma is a chronic disease, and the repetition of challenges during a lifetime characterizes this chronicity of Th2 eosinophilic inflammation [208]. The most used rodent models of asthma are those induced by OVA or aeroallergens, such as house dust mites [202]. These models have their own mechanistic uniqueness and some common mechanisms [209]. OVA can induce a robust airway allergic inflammation in mice, and notwithstanding, it is not a common inducer of allergic asthma in humans although it can mimic an antigen-driven type 2 immune response. On the other hand, house dust mites are a powerful allergen for humans [202,209,210,211]. This also leads to the conclusion that all animal models are important since they represent, at least in part, varied human conditions. The repetitive OVA airway exposure model (in combination with the prior immunization against OVA plus the adjuvant aluminum hydroxide) leads to an intense Th2 adaptive immune response with prominent eosinophilia, as well as hyperplasia of goblet cells and airway hyperresponsiveness, with additional well-characterized MHCI/II epitopes [209,212]. The house dust mite (e.g., *Dermatophagoides pteronyssinus* and *D. farinae*, the fungus *Alternaria alternata*, cockroach and pollen extracts) mechanisms include an intrinsic protease activity, favoring the start of the allergic inflammation, initially stimulating the alarmins production, ILC2 activation, and consequently a potent Th2 response. Interestingly, these asthma models do not need adjuvants, mimicking more reliably the natural exposure to airborne allergens via the nasal mucosa and airway tract for humans [209,211,213]. Representing the non-allergic asthma model, the adoptive transfer of polarized antigen-specific Th17 or mixed Th2/Th17 cells [214,215], pathogen recognition receptors (PPR)-bacterial lipopolysaccharide triggered non-eosinophilic airway inflammation or plasmid-containing allergen genetic information targeting dendritic cells to induce Th1 inflammation are also used [209,216,217,218].

Most of the data developing novel pharmaceutical forms for controlled release of flavonoids in asthma used the OVA model (Table 1). Therefore, the range of asthma models applied in the development and proof-of-concept of flavonoids’ therapeutic potential in asthma presents limitations. There is a need for testing these controlled release systems for flavonoid delivery in more varied disease models to gain a deeper understanding of the potential clinical applications of these pharmaceutical forms containing flavonoids, their limitations, and the most suitable disease conditions. Table 1 also highlights that most studies on flavonoids and controlled flavonoid release systems in asthma follow a similar standard of parameters to investigate the drug and pharmaceutical form activity, which means that similar physiopathological mechanisms were investigated in most studies. A suggestion to improve the understanding of the activity of flavonoids and pharmaceutical forms to control their release to support their therapeutic activity is to expand to unbiased methods. For instance, bulk RNA sequencing or single cell RNA sequencing and spatial transcriptomic analyses would improve the understanding of the mechanisms of controlled flavonoid release systems. The differences or lack of differences compared to current treatments would be clearer as well, as the extent of activity and shaping of the immune response in asthma. Searching for down-modulation of some groups of the main mechanisms is important and a valid approach; however, we see a window for improvement. As we mentioned earlier in the text, asthma symptoms are more prominent in adult women than in men [8,219]. However, many of the preclinical data using asthma models were developed using male mice and rats and used OVA for sensitization/challenges. It is also important to study the effects of flavonoids and their pharmaceutical forms in female rodents in addition to males, using aeroallergens (in which humans are naturally responsive, free of adjuvants) more consistently.

This section approached some possibilities of how to improve preclinical investigation and development that would potentially increase successful translation to humans. We would like to end this section by reinforcing that it contains some suggestions (possibly not an exhaustive list) to improve the field, and does not intend to invalidate what has been done. Making these possibilities feasible and performing the experiments is not as easy as making suggestions.

## 6. Conclusions

The present review highlights that there are few studies developing pharmaceutical forms for controlled release systems using flavonoids as active molecules for asthma treatment [140,141,142,143,144,145,146,147]. Not all flavonoids that are bioactive in asthma were investigated in terms of pharmaceutical development, which left open opportunities [102,112,117,118,120,153,165]. Flavonoids in general might not present the same side effects of corticoids since they do not bind and activate corticoid receptors, which is an advantage and would add them as an option when selecting the most adequate therapeutic approach to each patient (Section 5 and Table 2). There is a wide range of controlled release systems and routes of administration to improve the therapeutic efficacy of flavonoids in asthma that are not yet explored (Table 1 and Section 5). Investigating varied asthma disease models, sex dimorphism and deeper disease mechanisms studies also represent potential improvements in preclinical settings since these factors are relevant in disease context [219,220,221]. Taking these points into account might help to achieve better results in the phase of translating preclinical data to clinical investigation, as well as developing novel controlled release systems for flavonoid application in asthma therapy.

## Figures and Tables

**Table 1:** Summary of evidence found in studies evaluating the therapeutic potential of flavonoids in asthma models.

Flavonoid (Respective Class)	Dose (Route of Administration)	Type of Controlled Delivery System (Better or Not than Free Forms)	Asthma Model and Species (Observed Parameters)
Quercetin (flavonol) [140]	3 and 10 mg/kg (oral)	Microemulsions (yes for eosinophils recruitment)	OVA-induced asthma, BALB/c mice and Wistar rats (↓ eosinophils recruitment, NFκB p65, IL-4, IL-5, and mucus production)
Quercetin (flavonol) [141]	1 mg/kg (intra-peritoneal)	Nanocrystal (not evaluated)	OVA-induced asthma, BALB/c mice (↓ sIgE, IL-4, IL-5, and activation of mast cells)
Chafuroside A (flavone) [142]	0.1 mg/animal (oral)	Micelle (yes)	OVA-induced asthma, Sprague–Dawley rats (↓ eosinophils, macrophage, and neutrophils recruitment)
Bavachinin (flavanone) [143]	50 mg/kg (oral)	Nanoparticle (not evaluated)	OVA-induced asthma, BALB/c mice (↓ IL-4, IL-5, IL-13, and the % of cytokine-producing CD4^+^ T cells)
Bavachinin (flavanone) [144]	We did not have access to full manuscript	Nanosphere (not evaluated)	(↓ Th2 cytokines and inflammatory infiltrate)
Chrysin (flavone) [145]	50 mg/kg (oral)	Nanoparticle (yes)	OVA-induced asthma, BALB/c mice (↓ eosinophils, macrophage, neutrophils, and lymphocytes recruitment, IgE, NFκB p65, IL-4, IL-5, and IL-13)
Isoliquiritigenin (chalcone) [146]	5 and 10 mg/kg (intragastric)	Nanoemulsions (yes)	OVA-induced asthma, Sprague–Dawley rats (↓ eosinophils, neutrophils, and lymphocytes recruitment, sIgE, IL-4, and IL-5)
Baicalein (flavone) [147]	1 mg/mL (intranasal)	Loaded or emulsified nanoparticle (not evaluated)	OVA-induced asthma, BALB/c mice (↓ IL-5, mucus production, and AHR)

sIgE: specific IgE; AHR: airway hyperresponsiveness.

**Table 2:** Some of the flavonoids with therapeutic activity in asthma models to which controlled release systems could be developed.

Flavonoid (Respective Class)	Dose (Route of Administration)	Adverse Effects/Toxicity	Asthma Model and Species (Observed Parameters)
Naringenin (flavanone) [153]	20 and 40 mg/kg (oral)	Not evaluated	OVA-induced asthma, Wistar rats (↓ eosinophils recruitment, IL-4 and IL-13, and oxidative stress)
Kaempferol (flavonol) [154,155]	3, 30, and 90 mg/kg (subcutaneous) [154] 10 and 20 mg/kg (oral) [155]	Not evaluated in both	OVA-induced asthma, BALB/c mice (↓ eosinophils and total leukocyte recruitment, IL-5 and IL-13, ER-mediated stress response, AHR, and mucus production)
Fisetin (flavonol) [118,156]	1 and 3 mg/kg (intraperitoneal) [118] 0.3, 1, and 3 mg/kg (intravenous) [156]	Not evaluated in both	OVA-induced asthma, BALB/c mice (↓ adhesion molecules, eotaxin, TSLP, eosinophils, macrophages, neutrophils, and lymphocytes recruitment, NFκB p65, GATA-3, IL-33, IL-4, IL-5, and IL-13, AHR, and mucus production
Licochalcone A (chalcone) [157]	5 and 10 mg/kg (intraperitoneal)	33% mortality rate with 50 mg/kg dose	OVA-induced asthma, BALB/c mice (↓ eosinophils and lymphocytes recruitment, IgE, CCL11, IL-4, IL-5, and IL-13, AHR, and oxidative stress)
Luteolin (flavone) [158,159]	0.1, 1, and 10 mg/kg (intraperitoneal) [158] 0.1, 1, and 10 mg/kg (oral) [159]	Not evaluated in both	OVA-induced asthma, BALB/c mice (↓ GABA signaling, IgE, IL-4, IL-5, and IL-13, AHR, and mucus production)
Rutin (flavonol) [112]	7.5, 15, and 30 mg/kg (oral)	Not evaluated	OVA-induced asthma, Dunkin-Hartley guinea pig (↓ eosinophils and neutrophils recruitment, histamine, and AHR)
Apigenin (flavone) [117,160]	5 and 10 mg/kg (intraperitoneal) [117] 2 and 20 mg/kg (intraperitoneal) [160]	Not evaluated in both	OVA-induced asthma, BALB/c mice (↓ eosinophils recruitment, IgE, GATA-3, IL-4, IL-5, and AHR)
Hesperidin (flavanone) [120]	1 and 5 mg/kg (oral)	Not evaluated	OVA-induced asthma, BALB/c mice (↓ eotaxin, eosinophils recruitment, sIgE, GATA-3, IL-5, IL-17, and AHR)
Sakuranetin (flavanone) [102]	20 mg/kg (10 µL intranasal)	Not evaluated	OVA-induced asthma, BALB/c mice and Wistar rats (↓ eotaxin, eosinophils recruitment, sIgE, IL-5, NFκB, AHR, and oxidative stress)
Naringenin chalcone (chalcone) [161]	0.8 mg/kg (oral)	Not evaluated	OVA-induced asthma, BALB/c mice (↓ eosinophils recruitment, IL-4, IL-5, and IL-13, and AHR)
Genistein (isoflavone) [162]	20 and 40 mg/kg (intraperitoneal)	Not evaluated	OVA-induced asthma, BALB/c mice (↓ eosinophils and lymphocytes recruitment, GATA-3, STAT-6, IL-4, and IL-5)
Isoquercitrin (flavonol) [163]	15 mg/kg (oral)	Not evaluated	OVA-induced asthma, BALB/c mice (↓ eosinophils recruitment, and IL-5)
Galangin (flavonol) [164]	0.1 and 0.5 mg/kg (intraperitoneal)	Low toxicity with low dose (10 µM) in ASMCs human cells	OVA-induced asthma, BALB/c mice (↓ eosinophils and neutrophils recruitment, sIgE, TGF-β1, VEGF, and mucus production)
Sulfuretin (aurone) [165]	0.04 mg/kg (intraperitoneal)	Not evaluated	OVA-induced asthma, BALB/c mice (↓ eotaxin, eosinophils and lymphocytes recruitment, NFκB p65, IL-5, IL-3, and AHR)

ER: endoplasmic reticulum; AHR: airway hyperresponsiveness; TSLP: thymic stromal lymphopoietin; GATA-3: GATA binding protein 3; CCL11: CC motif chemokine ligand 11; GABA: gamma-amino butyric acid; sIgE: specific IgE; STAT-6: signal transducer and activator of transcription 6; ASMCs: airway smooth muscle cells; TGB-β1: transforming growth factor beta 1; VEGF: vascular endothelial growth factor.

## Data Availability

Not applicable.
